# Perioperatives Management der postmortalen Organspende: Anästhesie zwischen Ethik und Evidenz

**DOI:** 10.1007/s00101-021-01065-9

**Published:** 2021-11-08

**Authors:** Jan Sönke Englbrecht, Christian Lanckohr, Christian Ertmer, Alexander Zarbock

**Affiliations:** 1 Klinik für Anästhesiologie, operative Intensivmedizin und Schmerztherapie, Universitätsklinikum Münster, Albert-Schweitzer-Campus 1, Gebäude A1, 48149 Münster, Deutschland; 2 Antibiotic Stewardship (ABS)-Team, Institut für Hygiene, Universitätsklinikum Münster, Münster, Deutschland

**Keywords:** Organprotektive Maßnahmen, Anästhesiologisches Spendermanagement, Präkonditionierung, Irreversibler Hirnfunktionsausfall, Narkotika, Organ protective therapy, Anesthetic donor management, Preconditioning, Brain death, Narcotics

## Abstract

**Hintergrund:**

Die Anzahl postmortal gespendeter Organe ist in Deutschland weit geringer als der Bedarf. Dies unterstreicht die Wichtigkeit einer optimalen Versorgung während des gesamten Prozesses der Organspende.

**Fragestellung:**

Es existieren internationale Leitlinien und nationale Empfehlungen zu intensivmedizinischen organprotektiven Maßnahmen beim Organspender. Für das anästhesiologische Management fehlen evidenzbasierte Empfehlungen. Ziel dieser Übersichtsarbeit ist es, anhand der vorhandenen Evidenz die pathophysiologischen Veränderungen des irreversiblen Hirnfunktionsausfalls zu rekapitulieren und sich kritisch mit den empfohlenen Behandlungsstrategien und therapeutischen Zielgrößen auseinanderzusetzen. Auch auf ethische Aspekte der Betreuung des postmortalen Organspenders wird eingegangen.

**Methode:**

Diese Übersichtsarbeit basiert auf einer selektiven Literaturrecherche in *PubMed* (Suchwörter: „brain dead donor“, „organ procurement“, „organ protective therapy“, „donor preconditioning“, „perioperative donor management“, „ethical considerations of brain dead donor“). Internationale Leitlinien und nationale Empfehlungen wurden besonders berücksichtigt.

**Ergebnisse:**

Insgesamt ist die Evidenz für optimale intensivmedizinische und perioperative organprotektive Maßnahmen beim postmortalen Organspender sehr gering. Nationale und internationale Empfehlungen zu Zielwerten und medikamentösen Behandlungsstrategien unterscheiden sich teilweise erheblich: kontrollierte randomisierte Studien fehlen. Der Stellenwert einer Narkose zur Explantation bleibt sowohl unter pathophysiologischen Gesichtspunkten als auch aus ethischer Sicht ungeklärt.

**Schlussfolgerungen:**

Die Kenntnisse über die pathophysiologischen Prozesse im Rahmen des irreversiblen Hirnfunktionsausfalls und die organprotektiven Maßnahmen sind ebenso Grundvoraussetzung wie die ethische Auseinandersetzung mit dem Thema postmortale Organspende. Nur dann kann das Behandlungsteam in dieser herausfordernden Situation sowohl dem Organempfänger als auch dem Organspender und seinen Angehörigen gerecht werden.

## Abstract (en)

**Background:**

The number of organs donated after brain death in Germany is far lower than the demand. This underlines the importance of providing the brain-dead donor with optimal medical care throughout the donation process to decrease the risk of graft dysfunction. Several international guidelines and national recommendations guide the intensivists in organ-protective intensive care management of the brain-dead donor.

**Objective:**

The anesthetist is a key member during organ retrieval procedures and plays a crucial role in physiological donor management; however, evidence-based recommendations for the perioperative anesthetic management, drug treatment strategies and target values are lacking. Anesthesia literature about donor management is scarce and predominantly composed of reviews of practice, with little exploration of the scientific foundations. The aim of this review is to guide the anesthetist in the organ-protective perioperative therapy. The pathophysiological changes in patients who progress to brain death are briefly summarized. The available evidence, guidelines and expert opinions regarding medical treatment strategies and therapeutic goals in organ-protective therapy are reviewed. The ethical and pathophysiological considerations regarding the performance of anesthesia during organ retrieval are discussed.

**Methods:**

This review is based on a selective literature search in PubMed for publications regarding organ donation after brain death (keywords: “brain dead donor”, “organ procurement”, “organ protective therapy”, “donor preconditioning”, “perioperative donor management”, “ethical considerations of brain dead donor”). International guidelines, national recommendations and expert opinions were given special consideration.

**Results:**

Overall, the evidence for optimal perioperative organ-protective care of the brain-dead donor is limited. Most elements in the current recommendations and guidelines are based on pathophysiological reasoning, epidemiological observations or extrapolations from general organ-protective management strategies, and not on evidence from randomized controlled trials. National and international recommendations on treatment goals and drug therapy differ considerably in some aspects. The therapy concepts applied are very heterogeneous. Apart from medical challenges, the ethical circumstances are an additional burden for the entire treatment team. Whether anesthesia is reasonable during organ retrieval remains unclear. There is uncertainty about possible organ-protective effects of anesthetic drugs. Furthermore, ethical considerations raise the question of whether the determination of brain death and the use of anesthetic drugs during the procedure of organ retrieval are compatible with each other.

**Conclusion:**

Due to the lack of evidence, perioperative treatment should be guided by intensive care therapy strategies. The discussion about using anesthetic drugs during organ retrieval remains controversial. Pathophysiological considerations support the use of volatile anesthetics because of possible organ-protective effects. The use of neuromuscular blocking is justified to control any possible motor response resulting from spinal cord reflexes, whereas there is no evidence for a benefit from using opioids. Apart from that, it seems ethically problematic to anesthetise a brain-dead donor. Consequently, knowledge about the pathophysiological processes caused by brain death and about organ-protective therapy concepts are just as much a basic requirement as the consideration of ethical problems in organ donation after brain death. Only then are the caregivers able to do justice to both the organ recipient and the organ donor, as well as their relatives in this challenging situation.

Die Anzahl der Menschen in Deutschland, die auf ein Spenderorgan warten, ist weit größer als die Zahl der gespendeten Organe [15]. Ein potenzieller Organspender muss medizinisch optimal versorgt werden, um den Mangel an Organspenden nicht durch eine Organdysfunktion noch zu erhöhen [35]. Es fehlen allerdings evidenzbasierte Leitlinien und Empfehlungen für das organprotektive perioperative anästhesiologische Management. Zusätzlich ist der Stellenwert einer Narkose während der Explantation sowohl unter medizinischen als auch ethischen Aspekten unklar.

Um in Deutschland die Diskrepanz zwischen realisierten Organspenden und Menschen auf der Warteliste für eine Organtransplantation zu überwinden, gibt es erhebliche politische und gesellschaftliche Anstrengungen, um die Bereitschaft zur Organspende zu erhöhen [7]. Der Mangel an Spenderorganen bedeutet aber auch, dass die organprotektiven Maßnahmen beim postmortalen Organspender („donor after brain death“, DBD) besonders wichtig sind, um eine gute Transplantatfunktion sicherzustellen [35]. Die Organdysfunktion beim Spender ist einer der Hauptgründe, warum eine Transplantation trotz Zustimmung nicht realisiert werden kann [8, 43]. Den Intensivmedizinern stehen für die organprotektiven intensivmedizinischen Maßnahmen internationale Leitlinien [2, 27, 46] sowie nationale Empfehlungen zur Verfügung [13, 24, 29].

Kommt es nach irreversiblem Hirnfunktionsausfall (IHA) zu einer Organentnahme, hat das betreuende Anästhesie-Team u. a. die wichtige Aufgabe, die organprotektiven Maßnahmen fortzuführen. Überraschenderweise gibt es zum anästhesiologischen Management aber kaum evidenzbasierte Empfehlungen [8, 30]. Erschwerend kommt hinzu, dass aufgrund der geringen Spenderzahlen oft wenig Erfahrung mit Organentnahmen nach IHA vorhanden sein dürfte. Zum besseren Verständnis werden im Folgenden zunächst Grundprinzipien der organprotektiven Maßnahmen kurz zusammengefasst, und dann wird auf die anästhesiologischen Besonderheiten eingegangen.

## Organprotektive Maßnahmen beim Organspender

### Pathophysiologische Veränderungen im Rahmen des irreversiblen Hirnfunktionsausfalls

Der IHA führt infolge des Verlusts zentraler Regulationsmechanismen zu multifaktoriellen Organdysfunktionen. Diese betreffen v. a. die Hämodynamik, den Flüssigkeitshaushalt, die Lungenfunktion, die endokrinen Funktionen und die Gerinnung. Die zugrunde liegenden pathophysiologischen Veränderungen sind immer noch nicht komplett verstanden. Zur detaillierten Beschreibung der Vorgänge sei auf die entsprechende Literatur verwiesen [24, 27, 32, 46]. Eine Zusammenfassung der Empfehlungen zu organprotektiven Maßnahmen zeigt Tab. 1. Diese Empfehlungen sind abgeleitet aus den vorhandenen internationalen Leitlinien [2, 27, 32, 34, 46] und nationalen Empfehlungen [13, 24, 29], grundsätzlich ist jedoch die Evidenz für alle Empfehlungen aufgrund fehlender randomisierter kontrollierter Studien (RCT) gering [32, 35].

**Table 1:** 

Organprotektion	Zielwerte	Therapie	Bemerkung
Hämodynamisches Management	MAD ≥ 65 mm Hg	Norepinephrin (0,01–0,2 µg/kgKG und min) Vasopressin (0,5–2,4 IU/h)	Bei Hypotonie trotz adäquater Flüssigkeitssubstitution Vasopressin erwägen bei höheren NE-Dosierungen und/oder begleitendem DI
		Dobutamin (2–10 µg/kgKG und min)	Erwägen bei reduziertem HZV und bei persistierender Hypotonie trotz adäquater Flüssigkeitssubstitution und Vasopressortherapie Ggf. bei therapierefraktärer Bradykardie
		Esmolol, Urapidil, Metoprolol	Zur Therapie in der Phase des Katecholaminsturms
		Dopamin (4 µg/kgKG und min)	Als organprotektive Maßnahme ab Feststellung des IHA bis zur Explantation, insbesondere bei Hypotonie/Bradykardie ggf. Dosisreduktion bei Hypertonie und/oder Tachykardie
Pulmonales Management	TV: 6–8 ml/kg IBW p_a_O_2_/F_I_O_2_ ≥ 300 paO_2_/FIO_2_≥ 300 mm Hg pCO_2_: 35–45 mm Hg PEEP: 8–10 mm Hg	Lungenprotektive Beatmung	Keine routinemäßigen Recruitment-Manöver bei hämodynamisch instabilen Patienten Niedrigste F_I_O_2_ zum Erreichen einer adäquaten Oxygenierung (sO_2_ ≥ 95 %) möglichst niedriger „driving pressure (Spitzendruck – PEEP)“
Management von Flüssigkeit und Elektrolyten	Euvolämie Ausgeglichene Elektrolyte Medikamentöse Therapie des DI	Desmopressin (1–4 µg als Bolus i.v.) Vasopressin (0,5–2,4 IU/h)	Desmopressin bei DI, ggf. Repetitionsdosis (alle 2–4 h) Vasopressin bei hämodynamischer Instabilität
		Kristalloide, ggf. Glucoselösungen	Steuerung der Flüssigkeitsgabe mit dynamischen Variablen (PPV, V. cava Variabilität, TTE) Glucoselösungen bei therapierefraktärer Hypernatriämie
Endokrinologisches Management	Hormonsubstitution	T_3_ (4 μg Bolus, danach 3 μg/h i.v.) T_4_ (20 μg Bolus, danach 10 μg/h i.v.)	Bei hämodynamischer Instabilität oder EF < 45 % erwägen T_3_ bevorzugen bei gleichzeitiger Gabe von Hydrocortison
		Hydrocortison (300 mg/24 h i.v.)	Bei hämodynamischer Instabilität
Transfusions‑/Gerinnungsmanagement	INR: <1,5 PLT: ≥50.000/µl Hb ≥ 7 mg/dl	Ggf. Blut- und Gerinnungsprodukte (EK, FFP, TZ, Faktorensubstitution)	Therapie einer möglichen DIC obligat Medikamentöse Thromboseprophylaxe (bei fehlenden Kontraindikationen und normalen Gerinnungswerten)

*MAD* mittlerer arterieller Druck, *NE* Norepinephrin, *DI* Diabetes insipidus, *HZV* Herz-Zeit-Volumen, *IHA* irreversibler Hirnfunktionsausfall, *TV* Tidalvolumen, *IBW* Ideales Körpergewicht, *p*_*a*_*O*_*2*_ Sauerstoffpartialdruck, *F*_*I*_*O*_*2*_ inspiratorische Sauerstofffraktion, *pCO*_*2*_ Kohlendioxidpartialdruck, *PEEP* „positive endexspiratory pressure“, *PPV* Pulsdruckvariation, *TTE* transthorakale Echokardiographie, *T3* Trijodthyronin, *T4* Thyroxin, *EF* Ejektionsfraktion, *INR* International Normalized Ratio, *PLT* Thrombozyten, *Hb* Hämoglobin, *EK* Erythrozytenkonzentrat, *FFP* Fresh Frozen Plasma, *TZ* Thrombozytenkonzentrate, *DIC* disseminierte intravasale Koagulopathie

### Hämodynamisches Management

Das Management der hämodynamischen Instabilität ist sicherlich eine der größten Herausforderungen. Die Ursachen können mannigfaltig sein (intravasaler Volumenmangel, eingeschränkte Myokardfunktion, Vasoplegie etc.), und die Therapie ist entsprechend komplex. Es gibt keine Evidenz für ein optimales Monitoring-Verfahren zur Steuerung der hämodynamischen Therapie [32]. Eine invasive Blutdruckmessung, regelmäßige Kontrolle der Lactatwerte und wiederholte transthorakale Echokardiographien sind zweifellos sinnvolle und wenig invasive Maßnahmen [32]. Ob und wann ein erweitertes invasives hämodynamisches Monitoring indiziert ist, lässt sich anhand der vorhandenen Evidenz nicht beantworten [32].

Während im angloamerikanischen Raum als Zielwert ein mittlerer arterieller Druck (MAD) von 60–70 mm Hg empfohlen wird [32], fordert die Deutsche Stiftung Organtransplantation (DSO) einen vergleichsweise hohen MAD von 70–100 mm Hg [13] beim DBD. Bei kritisch kranken Patienten wird allgemein ein Ziel-MAD ≥65 mm Hg als ausreichend erachtet [5]. Aus Sicht der Autoren dieses Artikels gibt es keine evidenzbasierten Gründe, beim DBD einen höheren MAD anzustreben, auch in internationalen Leitlinien wird ein MAD ≥ 65 mm Hg als ausreichend erachtet [27, 34, 46]. Überhaupt erachten wir es als sinnvoll, bei den bisher nicht durch Evidenz belegten Aspekten der Behandlung des DBD die allgemeinen intensivmedizinischen Standards anzuwenden, sofern die spezifische Pathophysiologie nicht explizit andere Strategien erfordert.

Zum Erreichen des Ziel-MAD beim DBD wird im angloamerikanischen Raum Dopamin als Katecholamin der Wahl empfohlen, u. a. wegen der Erwägung, dass Norepinephrin die pulmonale kapilläre Permeabilität erhöht und die linksventrikuläre Nachlast negativ beeinflussen kann [27]. Für Dopamin konnte außerdem ein positiver Effekt auf die Organfunktion nach Herz- und Nierentransplantation gezeigt werden [37, 39]. Präklinische Untersuchungen sprechen zusätzlich für eine immunmodulatorische Wirkung, welche den oxidativen Stress abmildern kann [44]. Auch die DSO empfiehlt nach Feststellung des IHA die Gabe von Dopamin bis zur Explantation [14]. Dahingegen wird bei Patienten im septischen Schock Dopamin nicht generell empfohlen [5]. Es existieren Hinweise auf eine erhöhte Komplikationsrate und Letalität im Vergleich zu Norepinephrin [11]. Diese Diskrepanz ist zumindest teilweise durch die unterschiedliche zugrunde liegende Pathophysiologie erklärbar. Aus unserer Sicht kann Dopamin aufgrund der aktuellen Evidenzlage beim DBD angewendet werden, wobei RCT für eine generelle Empfehlung fehlen und letztlich die potenziell positiven Effekte sorgfältig gegen die potenziellen unerwünschten Nebenwirkungen (z. B. Arrhythmien) individuell abgewogen werden müssen [46]. Auch in internationalen Leitlinien ist die Empfehlung zur Anwendung von Dopamin nicht einheitlich [2, 32, 34], sodass die Anwendung letztlich eine Einzelfallentscheidung bleibt.

Sollte aufgrund der klinischen Evaluation ein Inotropikum indiziert sein, wird im deutschsprachigen Raum häufig Dobutamin empfohlen [13, 24], international eher Dopamin oder Epinephrin [32, 46]. Aufgrund fehlender RCT kann aber keine evidenzbasierte Empfehlung für ein bestimmtes Medikament ausgesprochen werden [32, 40, 46].

Zur kreislaufunterstützenden Therapie mit einem Vasopressor wird in Deutschland traditionell Norepinephrin empfohlen [13, 24]. Bei hohen Norepinephrindosierungen trotz adäquater Flüssigkeitssubstitution stellt Vasopressin wegen der V_1a_-Rezeptor-vermittelten Vasokonstriktion eine sinnvolle Ergänzung dar [46]. Studien zeigten nach Gabe von Vasopressin bei DBD eine signifikant schnellere Organerholung nach Lungentransplantationen und eine stabilere Nierenfunktion [36]. Da vergleichende RCT fehlen, ist allerdings unklar, ob die Gabe von Vasopressin alternativ oder zusätzlich zu Norepinephrin erfolgen sollte [32].

### Flüssigkeitstherapie

Neben der medikamentösen Kreislauftherapie stellt die adäquate Flüssigkeitssubstitution die zweite Säule der kreislaufunterstützenden Therapie dar. Zur Korrektur eines intravasalen Volumendefizits sollten bevorzugt balancierte Kristalloide oder bei einer begleitenden Hypernatriämie ggf. Glucoselösungen eingesetzt werden, sofern prophylaktische und kausale Maßnahmen gegen die Hypernatriämie ausgeschöpft sind [32]. Die von der DSO immer noch empfohlene 0,9 %ige Kochsalzlösung [13] ist aus unserer Sicht obsolet. Zur Flüssigkeitstherapie gibt es in Deutschland klare Empfehlungen, Kochsalzlösungen nicht zu verwenden [5]. Studien konnten zeigen, dass ein chloridrestriktives Regime das Auftreten einer höhergradigen akuten Nierenschädigung und die Notwendigkeit von Nierenersatzverfahren signifikant senkt [5].

Gegenüber einem früher propagierten liberalen Flüssigkeitsregime wegen einer vermeintlich besseren Nierentransplantatfunktion [32] wird inzwischen ein eher konservatives Vorgehen empfohlen. Nach Erreichen der Euvolämie sollte eine zusätzliche Flüssigkeitsüberladung vermieden werden [46]. Studien konnten für ein solches Vorgehen eine verbesserte Lungenfunktion ohne Beeinträchtigung der Nierenfunktion zeigen [32]. Zur Steuerung der Flüssigkeitsgabe sollte aus unserer Sicht nicht der zentrale Venendruck (ZVD), wie von anderen Autoren vorgeschlagen [13, 29], herangezogen werden. Dies wird beim kritisch kranken Patienten explizit nicht mehr empfohlen [5]. Dynamische Parameter (Schlagvolumenvariation, V.-cava-inferior-Variabilität etc.) eignen sich besser zur Steuerung der Flüssigkeitssubstitution [5, 46]. Deshalb ergeben sich unseres Erachtens keine Gründe, bei DBD von diesem Konzept abzuweichen.

Auch die Vorgabe einer Diuresemenge oder eines Ziellactatwertes, wie von anderen Autoren empfohlen [13, 29], erscheint uns nicht sinnvoll, insbesondere nicht, wenn diese nicht durch Handlungsempfehlungen bei Abweichungen von den Zielwerten ergänzt werden. Die Vorgabe einer zu erreichenden Diuresemenge könnte bei Unterschreitung zu einer unkritischen und inadäquaten liberalen Infusionstherapie führen, die nachweislich das Outcome einer akuten Nierenschädigung verschlechtert [48] und somit mutmaßlich mit einer verschlechterten Graft-Funktion einhergehen kann. Vielmehr sollte eine Oligurie, wenn möglich kausal therapiert werden. Das frühzeitige Entdecken und Vermeiden einer Polyurie als Zeichen eines beginnenden zentralen Diabetes insipidus (DI) ist sinnvoll und entsprechend zu therapieren (siehe Abschn. „Endokrinologisches Management“), da ein DI zu intravasaler Hypovolämie und Hypernatriämie führen kann, welche mit einer verschlechterten Transplantatfunktion assoziiert sind [35]. Im Rahmen einer Sepsis kann sich eine Gewebehypoperfusion in der Erhöhung des Serumlactatwertes manifestieren, weswegen bei kritisch kranken Patienten empfohlen wird, eine hämodynamische Stabilisierung mit dem Ziel einer Normalisierung der Lactatwerte zu steuern [5]. Vergleichbare Empfehlungen gibt es aufgrund fehlender Studien beim DBD nicht [45]. Sicherlich ist es sinnvoll, eine Laktacidose auch beim DBD frühzeitig zu erkennen und möglichst kausal zu therapieren. Die alleinige Vorgabe eines Zielwertes, der auch noch von den Zielwerten bei der Therapie der Sepsis abweicht, ist aus unserer Sicht aber nicht zielführend und wird weder von Evidenz gestützt noch von anderen Autoren empfohlen [45].

Spezifische Empfehlungen bezüglich eines Transfusionstriggers bei DBD existieren aufgrund fehlender RCT nicht. In Anlehnung an die Therapie bei kritisch kranken Patienten sollte ein Hämoglobinwert von 7 g/dl aber nicht unterschritten werden und die Indikation zur Transfusion immer auch unter Berücksichtigung der hämodynamischen Situation und evtl. Anzeichen einer Gewebshypoxie erfolgen [32].

### Pulmonales Management

Experimentelle Arbeiten und Metaanalysen legen nahe, dass eine lungenprotektive Beatmung die Zahl der erfolgreich transplantierten Lungen erhöhen kann [32]. Das respiratorische Management orientiert sich deshalb an den üblichen Standards zur lungenprotektiven Beatmung bei kritisch kranken Patienten [32, 46]. Es gibt keine Evidenz für einen Vorteil von alveolären Recruitment-Manövern; bei hämodynamisch instabilen DBD sollten diese unterbleiben [46].

### Endokrinologisches Management

Patienten mit IHA entwickeln durch Störung der hypothalamisch-hypophysären Steuerung komplexe Veränderungen des Wasser- und Elektrolythaushalts: häufig tritt ein DI auf [32]. Dieser sollte frühzeitig erkannt und adäquat therapiert werden. Neben Flüssigkeitsersatz mit kristalloiden Infusionslösungen empfiehlt sich hierzu eine Therapie mit Desmopressin. Desmopressin wirkt selektiv am V_2_-Rezeptor ohne signifikante vasopressorische Wirkung, sodass es bevorzugt zur Therapie des DI verwendet werden sollte, solange keine Hypotonie vorliegt [35]. Bei begleitender Hypotonie stellt Vasopressin eine sinnvolle Alternative dar [35, 46]. Vasopressin verbessert nicht nur V_1a_-Rezeptor-vermittelt die Vasoplegie, sondern über V_2_-Rezeptoren im distalen Konvolut des Tubulus und des Sammelrohrs der Niere den Einbau von Aquaporinen und bewirkt eine Steigerung der Wasserreabsorption [35]. Bei frühzeitiger Gabe kann so die Entwicklung eines DI abgemildert werden [29, 35, 46]. Evidenzbasierte Empfehlungen zur supportiven Therapie der Hypernatriämie bei IHA mit enteraler Zufuhr von freiem Wasser existieren nicht. Aus pathophysiologischen Überlegungen und in Anlehnung an die Therapie des zentralen DI bei wachen Patienten erscheint die Gabe allerdings sinnvoll, soweit keine Kontraindikationen für eine enterale Gabe vorliegen [10, 34].

Potenzielle DBD zeigen häufig erniedrigte Spiegel von Trijodthyronin (T_3_). Ursprünglich wurde dies mit einer hypothalamisch-hypophysären Dysfunktion erklärt, jedoch zeigten Studien eine erhaltene Funktion der Adenohypophyse mit erniedrigten bis erhöhten Spiegeln des thyreoidstimulierenden Hormons (TSH) [23, 25, 27]. Thyroxin (T_4_) und reverses T_3_ (rT_3_) sind ebenfalls häufig normal oder erhöht [32]. Diese Konstellation deutet darauf hin, dass es sich eher um eine Störung wie bei kritisch kranken Patienten handelt („euthyroid sick syndrome“) [35]. Die Studienlage zur Substitution von Schilddrüsenhormonen beim DBD ist entsprechend nicht einheitlich [27]. Teilweise wird sie in internationalen Leitlinien empfohlen [27], andere empfehlen sie nicht [20, 34, 46] bzw. geben keine eindeutige Empfehlung ab [2]. Im Falle einer therapierefraktären Hypotension bzw. einer reduzierten linksventrikulären Ejektionsfraktion trotz optimierter hämodynamischer Therapie scheint ein Therapieversuch aus unserer Sicht jedoch gerechtfertigt, zumal es kaum Hinweise gibt, dass die Anwendung negative Effekte auf die Transplantatfunktion haben könnte [29, 32, 35]. Während die Applikation von T_3_ bzw. T_4_ als gleichwertig empfohlen wird [27], erscheint aufgrund der Pathophysiologie (höhere Potenz, keine Konversion von T_4_ zu T_3_ erforderlich) und der häufig parallel erfolgten Gabe von Kortikosteroiden die Gabe von T_3_ sinnvoller [35]. Alternativ kann T_4_ auch enteral verabreicht werden, falls i.v.-Präparate nicht verfügbar sind und keine Hinweise für eine mangelnde enterale Resorption vorliegen [35].

Postuliert werden weiterhin eine Störung der Hypothalamus-Hypophysen-Nebennieren(HPA)-Achse und ein Anstieg proinflammatorischer Zytokine, welche die Organdysfunktionen weiter aggravieren [29]. Für Ersteres gibt es allerdings wenig Evidenz, da ähnlich wie bei den Schilddrüsenhormonen die HPA-Achse normalerweise nicht gestört ist [23, 25, 32] und eine hämodynamische Instabilität nicht mit einer Hypocortisolämie oder fehlender Kortikotropinantwort der Nebennieren assoziiert ist [25, 35]. Für eine HPA-Dysfunktion, die zu reduzierten ACTH- und Cortisolspiegel beim IHA führt, gibt es somit aus evidenzbasierter Sicht nicht genug Hinweise, um DBD regelhaft aus diesen Gründen mit Kortikosteroiden zu behandeln [35]. Für Zweiteres sprechen Beobachtungen, die eine verbesserte Lebertransplantatfunktion [28] bzw. bessere Oxygenierung und erhöhte Rate an Lungentransplantationen [22] nach Gabe von Kortikosteroiden zeigten. Dies konnte bisher aber durch andere Studien nicht bestätigt werden; auch die optimale Dosierung bleibt unklar [32, 35]. In einer systematischen Übersichtsarbeit kommen Dupuis et al. zu dem Schluss, dass die Evidenz zur routinemäßige Anwendung von Kortikosteroiden bei DBD widersprüchlich und von nichtausreichender Qualität ist [18]. Von der DSO wird bei IHA ein Bolus von 250 mg Methylprednisolon mit anschließender kontinuierlicher Zufuhr von 100 mg/h empfohlen [13]. Internationale Leitlinien empfehlen die Gabe (in ähnlichen Dosierungen) nur bei hämodynamisch instabilen Patienten [2, 27, 32]. Hinsichtlich des Transplantationsergebnisses von Herz und Lungen waren hohe Dosierungen gegenüber einer „Low-dose“-Therapie (300 mg Hydrocortison/24 h) nicht überlegen [17]. Zusammenfassend besteht aus unserer Sicht keine ausreichende Evidenz für eine routinemäßige Anwendung von Kortikosteroiden und bei der Entscheidung zur Gabe eine Dosierung von 300 mg/24 h Hydrocortisonäquivalent zu überschreiten [18, 35, 46].

### Gerinnungsmanagement

Der IHA kann zu einer Aktivierung der Blutgerinnung führen. Die Inzidenz einer disseminierten intravasalen Koagulopathie (DIC) wird mit 15–25 % angegeben [32]. Die Behandlung einer möglichen DIC ist obligat und erfolgt analog zur Therapie bei anderen kritisch kranken Patienten. Zur Organexplantation sollte die Gerinnung optimiert werden [32].

Explizite Empfehlungen zur Thromboseprophylaxe beim DBD gibt es nicht. Wegen des prokoagulatorischen Zustands scheint aber bei fehlenden Kontraindikationen eine medikamentöse Thromboseprophylaxe sinnvoll [32].

## Perioperatives anästhesiologisches Management

### Perioperative Organprotektion

Obwohl spezifische Empfehlungen für die perioperative Phase fehlen [40], erscheint es sinnvoll, die Zielwerte und die bereits eingeleiteten organprotektiven Maßnahmen im OP bis zur Organentnahme fortzuführen [40]. Eine Zusammenfassung unserer Empfehlungen, die aus der vorhandenen (niedrigen) Evidenz und Expertenmeinungen extrapoliert wurden, zeigt Tab. 2.

**Table 2:** 

Empfehlungen	Maßnahmen	Bemerkung
Organprotektive Maßnahmen	Fortführung der intensivmedizinischen Maßnahmen zur Organprotektion	Auf der Intensivstation begonnenes Monitoring sowie medikamentöse Therapie und Zielwerte grundsätzlich während der Explantation beibehalten
Anästhesie zur Explantation	Volatile Anästhetika (z. B. Sevofluran 1 MAC) Muskelrelaxation	Volatile Anästhetika zur organprotektiven Therapie Muskelrelaxanzien zur Optimierung des chirurgischen Eingriffs Opioide aus evidenzbasierter Sicht nicht notwendig
Flüssigkeitsmanagement	Kristalloide Infusionslösungen	Vermeidung von Hypo- und Hypervolämie
Transfusionsmanagement	EK, ggf. Gerinnungsprodukte	Hb ≥ 7 mg/dl Gabe von Gerinnungsprodukten nach Klinik und Labordiagnostik
PAP	Nach Klinikstandard (z. B. Cephalosporin 1./2. Generation, Repetition nach 2 HWZ)	PAP oder Fortführung der intensivmedizinisch begonnenen Antibiotikatherapie

*MAC* minimale alveoläre Konzentration, *EK* Erythrozytenkonzentrat, *Hb* Hämoglobin, *PAP* perioperative Antibiotikaprophylaxe, *HW*Z Halbwertszeit

### Perioperative Antibiotikaprophylaxe

Der Effekt einer Antibiotikaprophylaxe ist die Keimreduktion im Operationsgebiet, um postoperative Wundinfektionen zu verhindern. Die systemische Antibiotikagabe ergänzt in diesem Zusammenhang die „üblichen“ operativen Maßnahmen der Asepsis und Antisepsis. Es erscheint naheliegend, dass eine bakterielle Kontamination explantierter Organe vermieden werden sollte, da dies wiederum Wundinfektionen beim Organempfänger begünstigen kann. Dieses Anliegen reflektiert sich in Empfehlungen zur Antibiotikaprophylaxe bei Empfängern einer Transplantation solider Organe [1]. In diesem Zusammenhang erscheinen alle Maßnahmen sinnvoll, die eine intraoperative Keimreduktion unterstützen. Daher empfehlen wir die Gabe einer perioperativen Antibiotikaprophylaxe (PAP) zur Explantation. Diese folgt den gängigen Standards [4]. Erhält ein DBD aus anderer Indikation ein Antibiotikum, sollte diese Therapie bis zum Abschluss der Explantation fortgesetzt werden. In diesen Situationen ist eine zusätzlich PAP in der Regel unnötig.

### Anästhesie zur Organexplantation

Während einer Operation werden Hypnotika, Analgetika und ggf. Muskelrelaxanzien verwendet, um einen reversiblen Bewusstseinsverlust, eine Analgesie und eine Unterdrückung vegetativer und motorischer Reflexe zu erreichen. Diese Beschreibung macht deutlich, dass sich die Aufgaben des Anästhesie-Teams im Rahmen einer „normalen“ Operation deutlich von denen einer postmortalen Organexplantation unterscheiden. Der DBD hat per definitionem kein Bewusstsein und Schmerzempfinden mehr. Dies legt den Schluss nahe, dass lediglich die Gabe von Muskelrelaxanzien zur Unterdrückung spinaler motorischer Reflexe erforderlich sein könnte.

Sehr pragmatisch sehen dies auch der Wissenschaftliche Beirat der Bundesärztekammer und der Deutsche Ethikrat. Weil nach IHA sowohl Bewusstsein als auch das Schmerzempfinden erloschen sind, sehen sie keine Notwendigkeit für die Durchführung einer Narkose [6, 16]. Wenig überraschend hat diese Sichtweise in der Vergangenheit sowohl in der Laienpresse [31] als auch in Fachzeitschriften zu teils emotionalen Reaktionen geführt [38].

Diese teilweise öffentlich kontrovers geführte Diskussion könnte nicht zuletzt auch Angehörige von DBD verunsichern. Häufig wird das Behandlungsteam mit der Frage konfrontiert, ob der Organspender auch wirklich nichts während der Entnahmeoperation spürt, und es wird der Wunsch geäußert, die Explantation unter Narkose durchzuführen [9]. Ein zu sorgloser oder unreflektierter Umgang mit diesem Anliegen könnte das Vertrauen der Öffentlichkeit in das Konzept der postmortalen Organspende gefährden [26]. Zusätzlich könnte die Gabe von Anästhetika implizieren, dass der DBD wie eine lebende Person behandelt wird, was ethisch problematisch ist [26]. Eine tiefergehende Diskussion der ethischen Problematik würde den Rahmen dieser Übersichtsarbeit sprengen, es sei auf die entsprechende Literatur verwiesen [26, 47]. Im Folgenden soll aber auf medizinische Erwägungen zur Gabe von Anästhetika eingegangen werden.

### Hypnotika

Auf einen chirurgischen Stimulus reagieren DBD mit einem Anstieg von Herzfrequenz und Blutdruck, was mit erhaltenen Reflexen auf spinaler Ebene erklärt wird [40]. Hieraus leitet sich die Überlegung ab, dass Hypnotika zur Unterdrückung hämodynamischer Reaktionen während der Explantation organprotektive Effekte haben könnten [19, 30, 40]. Zusätzlich konnte für volatile Anästhetika im Tiermodell und bei herzchirurgischen Patienten ein organprotektiver Effekt durch Verminderung eines Ischämie-Reperfusion-Schadens gezeigt werden [12, 19, 41]. Postuliert wird dabei eine pharmakologische Präkonditionierung („anaesthetic preconditioning“), welche über verschiedene intrazelluläre Mechanismen vermittelt wird (für Details: [12, 41]). Im Tiermodell konnte mit 1 MAC (minimale alveoläre Konzentration) Sevofluran ein experimenteller Myokardinfarkt signifikant reduziert werden [42]. Im Lungentransplantation-Tier-Modell führte die Gabe von 1 MAC Sevofluran zu einer besseren Oxygenierung und geringeren Inflammationsreaktion [3]. Eine klinische Arbeit konnte eine bessere Lebertransplantatfunktion zeigen, wenn im Rahmen der Explantation 2 % Sevofluran endtidal verabreicht wurden [33]. Generell ist die Evidenzlage für den Einsatz volatiler Anästhetika während der Explantation gering, sodass keine eindeutige Empfehlung ausgesprochen werden kann [30, 40]. Aus unserer Sicht ist deren Anwendung zur Organexplantation gerechtfertigt; auch andere Autoren sprechen sich dafür aus [19, 30, 47]. Dies darf aber nicht als Beleg dafür missinterpretiert werden, dass dem Konzept des IHA misstraut wird, sondern vielmehr, dass diese Medikamente zur Organprotektion eingesetzt werden, völlig unabhängig von ihren Auswirkungen auf den Spender [40]. Anders formuliert: Die Narkotikaanwendung ist nicht intendiert als Behandlung des Organspenders, sondern als organprotektive Maßnahme für den Organempfänger [19, 30].

### Opioide

Trotz der problematischen ethischen Implikationen ist es ein gängiges Konzept, zur Organexplantation Opioide zu verabreichen [8], wobei die Evidenz für einen organprotektiven Effekt fehlt [40]. Die Gabe von Fentanyl im Rahmen der Explantation führt zu keiner Reduktion endogen freigesetzter Katecholamine [21], auch hat die Gabe keinen Effekt auf Herzfrequenz oder Blutdruck [30]. Deshalb äußern einige Autoren, dass Opioide im Rahmen der Organentnahme nicht appliziert werden sollten [40]. Andererseits gibt es aber keine Hinweise, dass die Gabe organschädigend wäre [26]. Unter pathophysiologischen Erwägungen ist die Gabe von Opioiden nicht notwendig und widerspricht auch dem Konzept des IHA. Zwar fällt das Vorenthalten von Schmerzmitteln in dieser Situation vielen Kolleginnen und Kollegen in der Praxis verständlicherweise schwer, aber unter rein medizinischen Gesichtspunkten kann aus unserer Sicht keine Empfehlung für die Gabe von Opioiden zur Explantation ausgesprochen werden.

### Muskelrelaxanzien

Durch den IHA geht die hemmende Wirkung des Hirnstamms auf das Rückenmark verloren. Somatisch und viszeral ausgelöste Reize werden deshalb mit überschießenden spinalen Reflexen beantwortet [40]. Neuromuskuläre Reflexe können sich neben hämodynamischen Veränderungen auch als spontane Bewegungen äußern, welche für das anwesende Personal sehr belastend sind [30]. Um diese Reflexbewegungen zu unterbinden und zur Schaffung optimaler Entnahmebedingungen sollte daher eine Muskelrelaxierung während der Explantation durchgeführt werden [40].

## Fazit für die Praxis

- Kenntnisse über die pathophysiologischen Vorgänge beim irreversiblen Hirnfunktionsausfall sind Grundvoraussetzung für eine adäquate perioperative Therapie.
- Aufgrund fehlender randomisierter kontrollierter Studie (RCT) ist die Evidenz bezüglich der perioperativen organprotektiven Maßnahmen zur postmortalen Organentnahme gering; sie sollte sich an den etablierten intensivmedizinischen Konzepten orientieren und perioperativ fortgesetzt werden.
- Eine perioperative Antibiotikaprophylaxe wird empfohlen.
- Volatile Anästhetika haben möglicherweise ergänzende organprotektive Effekte, ihre Anwendung ist dabei nicht intendiert als Narkose für den Organspender, sondern als organprotektive Maßnahme für den Organempfänger.
- Es gibt keine evidenzbasierten Hinweise für einen organprotektiven Effekt von Opioiden während der Explantation.
- Zur Unterdrückung spinaler Reflexe und zur Optimierung der Entnahmebedingungen sollte eine Muskelrelaxation erfolgen.
